# An international survey of physicians regarding clinical trials: a comparison between Kyoto University Hospital and Seoul National University Hospital

**DOI:** 10.1186/1471-2288-13-130

**Published:** 2013-10-25

**Authors:** Toshiko Ito-Ihara, Jeong-Hwa Hong, Ock-Joo Kim, Eriko Sumi, Soo-Youn Kim, Shiro Tanaka, Keiichi Narita, Taichi Hatta, Eun-Kyung Choi, Kyu-Jin Choi, Takuya Miyagawa, Manabu Minami, Toshinori Murayama, Masayuki Yokode

**Affiliations:** 1 Department of Clinical Innovative Medicine, Institute for Advancement of Clinical and Translational Science, Kyoto University Hospital, 54 Kawaharacho, Shogoin, Sakyo-ku, Kyoto, Japan; 2 Department of the History of Medicine and Medical Humanities, Seoul National University College of Medicine, 103 Daehak-ro, Jongno-Gu, Seoul, South Korea; 3 Department of Pharmacoepidemiology, Graduate School of Medicine and Public Health, Kyoto University, Yoshida-Konoe-cho, Sakyo-ku, Kyoto, Japan; 4 Institute of Medical History and Culture, Seoul National University Hospital, 101 Daehak-ro, Jongno-gu, Seoul, South Korea; 5 Program in History and Philosophy of Science, Seoul National University, 1 Gwanak-ro, Gwanak-gu, Seoul, South Korea

**Keywords:** Attitude of physicians, Clinical trials, Questionnaire, Japan, South Korea

## Abstract

**Background:**

International clinical trials are now rapidly expanding into Asia. However, the proportion of global trials is higher in South Korea compared to Japan despite implementation of similar governmental support in both countries. The difference in clinical trial environment might influence the respective physicians’ attitudes and experience towards clinical trials. Therefore, we designed a questionnaire to explore how physicians conceive the issues surrounding clinical trials in both countries.

**Methods:**

A questionnaire survey was conducted at Kyoto University Hospital (KUHP) and Seoul National University Hospital (SNUH) in 2008. The questionnaire consisted of 15 questions and 2 open-ended questions on broad key issues relating to clinical trials.

**Results:**

The number of responders was 301 at KUHP and 398 at SNUH. Doctors with trial experience were 196 at KUHP and 150 at SNUH. Among them, 12% (24/196) at KUHP and 41% (61/150) at SUNH had global trial experience. Most respondents at both institutions viewed clinical trials favorably and thought that conducting clinical trials contributed to medical advances, which would ultimately lead to new and better treatments. The main reason raised as a hindrance to conducting clinical trials was the lack of personnel support and time. Doctors at both university hospitals thought that more clinical research coordinators were required to conduct clinical trials more efficiently. KUHP doctors were driven mainly by pure academic interest or for their desire to find new treatments, while obtaining credits for board certification and co-authorship on manuscripts also served as motivation factors for doctors at SNUH.

**Conclusions:**

Our results revealed that there might be two different approaches to increase clinical trial activity. One is a social level approach to establish clinical trial infrastructure providing sufficient clinical research professionals. The other is an individual level approach that would provide incentives to encourage doctors to participate in and conduct clinical trials.

## Background

Industry-sponsored clinical trials have traditionally been carried out in North America, Western Europe, and Oceania. More recently, there has been an increase in the globalization of clinical trials by the pharmaceutical industry, especially for biological products [1]. Reasons cited for this shift include the ability to reduce operational costs while recruiting a large number of patients in a timely manner, the establishment of contact research organizations focused on global clinical trials, the expansion of market size, increased research capacity in emerging countries, improvements in regulatory development, and the harmonization of guidelines for clinical practice and research [1]. Thus, international clinical trials are now rapidly expanding into emerging countries such as those in Eastern Europe, South America, and Asia.

In compliance with the International Conference on Harmonisation - Good Clinical Practice GCP (ICH-GCP) in 1996 [2], the Japanese government established a ministerial ordinance on GCP in 1997 [3], which was implemented in April of 1998. However, failure to provide a proper infrastructure led to the continuous decrease in domestic clinical trials with many of them being outsourced abroad. The 160 Japanese clinical trials held in 1993 plummeted to just 43 in 2001. With Japan being left out of global clinical trials/global development, this came to be known as the “hollowing out of clinical trials in Japan”. As a consequence, Japan has been experiencing a “Drug/Device Lag” phenomenon, where there is a delay in the approval of new drugs and devices that are already approved in the US and in European countries [4,5]. To improve this negative situation, the Ministry of Health, Labour and Welfare (MHLW) of Japan has revamped their clinical trial consultation system to establish an environment more conducive to clinical trials and to enhance the participation of Japan in global clinical trials since 2003. The Japanese government launched *a 3-year* (2003-2006) and *a new 5-year clinical trial activation plan* (2007-2011) [6]. Improvements in policies to greatly strengthen the review system have also been initiated [7]. Owing to these measures, the number of investigated new drug (IND) applications in Japan increased from 361 in 2003 to 530 in 2007, 495 in 2008, and 553 in 2009 [8], and the notorious drug lag has been cut in Japan from 3.4 years in 2007 to 2 years in 2009, shrinking the approval time of new drugs by almost half [9]. However, the proportion of global trials in Japan still remains relatively low compared with other Asian countries: 7.5% of the total in 2007, 15.6% in 2008, and 20.2% in 2009 [10].

In contrast, the number of domestic and global clinical trials has been rapidly increasing in South Korea following the introduction of the Korean (K)-GCP in 1996 leading to the separation of new-drug applications (NDAs) and INDs in 2000. In accordance with ICH-GCP, a new K-GCP was implemented in 2001 [11,12]. These changes have all led to *a Regional Clinical Trial Centers (CTC) funding program* supported by the Korean Ministry for Health, Welfare and Family Affairs, which has aided in the establishment of global standard GCP CTCs in South Korea. In December 2007, *the Regional CTC funding program* was expanded by the South Korean government to meet the increasing demand for clinical trials and *the Korea National Enterprise for Clinical Trials (KoNECT)* was established. As a result, the number of multinational sponsored clinical trials in South Korea has dramatically increased from 5 in 2000 to 148 in 2007 and 216 in 2008, to the point where global trials have exceeded the number of domestic clinical trials [13].

Several studies have been performed to obtain a better idea of Japanese physicians’ attitudes towards clinical research [14,15]. Yanagawa et al. surveyed 89 doctors in 2000 and 62 doctors in 2004 at Tokushima University Hospital, Japan [14]. Sumi et al. surveyed 310 physicians in 2007 at Kyoto University Hospital (KUHP), Japan [15]. Although the attitude towards participating in clinical research was favorable, they felt that physicians faced several barriers when initiating clinical studies such as cumbersome paperwork and time constraints [14,15]. To date, similar surveys regarding doctors’ attitudes toward clinical research/clinical trials in South Korea have not been reported.

It is matter of considerable concern that the growth in the number of clinical trials in Japan and South Korea has been different, despite implementation of similar government-supported measures. The difference in the clinical trial environment of each country might influence the respective physicians’ attitudes and experience. Therefore, we designed a uniform questionnaire survey to explore how individual physicians perceive the issues surrounding clinical trials in two university hospitals in Japan and South Korea: KUHP and Seoul National University Hospital (SNUH). Elucidation of these differences may help facilitate recruitment of physicians in future trials and thereby, help develop a better environment for conducting clinical trials.

## Methods

### Study design

We conducted a questionnaire survey among physicians in KUHP and SNUH between September and December 2008. Kyoto University had been designated as one of seven distinguished research sites to participate in *the Coordination, Support and Training Program for Translational Research* between 2007 and 2011, and most recently *the Second Stage Network Program for Accelerating Translational Research* from 2012 through 2017 by the Ministry of Education, Culture, Sports, Science and Technology (MEXT) Japan. A total of 77 industry-sponsored trials approved by the KUHP Institutional Review Board (IRB) were conducted in 2008, of which 14% were multinational and 1.3% were Phase I trials. A total of 439 clinical studies were approved by the KUHP ethics committee in 2008 and of those ~100 were investigator-initiated trials, which were not aimed at drug approval.

SNUH comprises of a Main Hospital, Children’s Hospital, Cancer Hospital, and the Biomedical Research Institute and is one of the largest university hospitals in South Korea. SNUH has been designated as one of the qualified clinical trial facilities by the governmental funding of *the Regional CTC Program* since 2004 and the *KoNECT Program* since 2007. In SNUH, a total of 218 clinical trials were conducted in 2008, of which 37% were multinational and ~20% were Phase I trials. ~100 investigator-initiated clinical trials were also approved by the SNUH-IRB in 2008.

To optimize the response rate of the questionnaires, we informed the directors of each of the 36 departments in KUHP and 18 departments in SNUH to explain the study protocol and to ask for their participation in the study. Each department representative distributed the study description and questionnaire to all physicians belonging to the department. Faculty, fellows, staff doctors (full time physicians), residents, and doctoral students with medical degrees were invited to participate. Approvals by The Kyoto University Graduate School And Faculty of Medicine Ethics Committee and The Seoul National University College of Medicine And Seoul National University Hospital Institutional Review Board were obtained in July 2008 as E-489 and June 2008 as C-0806-050-247, respectively.

### Development of questionnaire

We created new survey questions based on previously conducted surveys regarding clinical studies for physicians [14,15].

In a previous study, Yanagawa et al conducted an opinion survey for physicians at Tokushima University regarding their experience with industry. The aim of their study was to explore the view of physicians towards clinical trials for drug development, in order to improve communication among participants, sponsors, and investigators. The questionnaire started with asking the physicians about their experience in conducting industry-sponsored trials following the introduction of GCP in Japan in 1997. Using 4 point scale, this was then followed by questions aimed at revealing the general attitude of the physicians towards industry-sponsored trials. Furthermore, the investigators were asked if they had faced any difficult situations during the industry-sponsored trial in several subordinate items with multiple choice answers. The investigators were also asked if they found merit in participating in industry-sponsored trials through an open-ended question.

In another study, Sumi et al conducted a survey at KUHP to investigate the willingness of physicians to participate in clinical research. The aim of their study was to identify methods of support and training that assist physicians in conducting clinical research. The target of interest of the survey was “clinical research” which not only included industry-sponsored clinical trials and academia-driven trials, but also “general clinical research conducted by physicians” such as observational studies and epidemiological studies. They inquired about the merits of participating in clinical research and about the difficulties faced in conducting such research using 5 and 9 multiple choice subordinate items, respectively.

Our questionnaire survey for this study took into consideration the past two surveys and focused on clinical trials (including trials conducted by industry as well as academia). In light of the recent increase in multi-national clinical trials, the items related to global trials were asked separately. The term “clinical trial” used in this survey was not categorized by study design or funding body and thus, the definition of “clinical trial” in this survey included all randomized controlled trials, non-randomized trials, pharmaceutical-sponsored trials, and investigator-initiated trials. We inquired through a 4 tier scale about the merits of participating in clinical trials (Question #8) and about the difficulties faced in conducting such trials (Question #10) using 10 and 11 subordinate items respectively. Furthermore, regarding multi-national clinical trials sponsored by a global pharmaceutical company, we inquired if they had experience in participating in such a trial and if so, then through an 8 subordinate-item question via a 4 tier scale asked about the major obstacles in participating in a multi-national trial (Question #16). The questionnaire was first constructed in English and subsequently translated into Japanese and Korean before distribution at both institutions. The questionnaire was anonymous and contained 5 pages in English, 5 pages in Japanese, and 4 pages in Korean. The questionnaire is available online (see Additional file 1).

### Statistical analysis

Data were summarized as the frequency of each answer and their proportion. Each subordinate item in Questions #8, 10, 14 and 16 was treated as a binary variable for statistical analysis. Fisher’s exact tests were used for the comparison between hospitals. All reported p values are two-tailed, and p < 0.01 was taken to indicate statistical significance. Statistical analysis was performed using SAS Version 9.2 (SAS Institute, Cary, NC, USA).

### Supplemental analysis on the qualitative portion of the questionnaire

Categorization of the answers from the open-ended questions (Question #9 and #17) were performed in following manner. First, a researcher (TI) and an ethics researcher (TH) extracted pieces of comments from all transcripts. Then, under the supervision of a psychologist (KN), the researchers carefully conceptualized and categorized the attributes from the comments and created a definition of each category for all the attributes.

## Results

### Characteristics of respondents and their interest level and experience in clinical trials

There were a total of 1,091 physicians in KUHP at the time of the survey (345 faculty, 181 staff doctors, 133 junior residents, and 432 PhD students in the department of clinical medicine). There were a total of 1,661 doctors at SNUH at the time of the survey (420 faculty, 257 fellows, 777 residents, and 207 interns).

34 out of 36 departments in KUHP and all 18 departments in SNUH consented to participate in this survey. We were not able to get any response from the directors of the Department of Endocrinology and Metabolism and the Department of Medical Informatics at KUHP for reasons not known to us. The departments consisted of 11 faculty members and 8 staff doctors in total at the time of the survey.

Among 471 physicians from the 34 departments at KUHP, a total of 301 (64%) completed the questionnaire. Among 882 physicians from the 18 departments in SNUH, a total of 398 (45%) completed the questionnaire. Table 1 presents the characteristics of respondents and their interest level and experience in clinical trials. In KUHP, 10%, 52%, 32%, and 6% of respondents were in their 20s, 30s, 40s, and over 50, respectively. Females represented 17% (50/301) of KUHP respondents. A total of 169 faculty (56%) and 132 non-faculty (staff doctors, residents and PhD students) members responded. In SNUH, 44%, 42%, 8%, and 7% of respondents were in their 20s, 30s, 40s, and over 50, respectively. Females represented 37% (147/398) of all SNUH respondents. A total of 90 faculty (23%) and 308 non-faculty (fellows, residents and PhD students) members responded. In KUHP, faculty members were more likely to complete the survey than non-faculty members and the converse was true at SNUH. There were a greater proportion of young physicians answering the survey at SNUH compared to KUHP.

**Table 1:** Demographic characteristics of the respondents (N = 699)

		**KUHP**			**SNUH**		
		**Doctors with clinical trial experience**	**Doctors without clinical trial experience**	**Total**	**Doctors with clinical trial experience**	**Doctors without clinical trial experience**	**Total**
		**% (n)**	**% (n)**		**% (n)**	**% (n)**	
		**(196)**	**(104)**	(301)*	**(150)**	**(242)**	(398)*
Age range							
	<=29	6 (12)	17 (18)	10 (30)	19 (29)	59 (143)	44 (175)
	30-39	44 (86)	66 (69)	52 (156)	47 (70)	39 (94)	42 (166)
	40-49	42 (83)	13 (14)	32 (97)	18 (27)	1 (2)	8 (30)
	> = 50	8 (15)	3 (3)	6 (18)	16 (24)	1 (3)	7 (27)
Gender (Female ratio)		13 (25)	24 (25)	17 (50)	25 (38)	45 (109)	37 (147)
Faculty		70 (138)	30 (31)	56 (169)	51 (76)	5 (13)	23 (90)
Majority of Doctors							
	Internal Medicine**	36 (71)	30 (31)	34 (102)	23 (35)	16 (38)	19 (75)
	Surgery***	21 (41)	11 (11)	17 (52)	3 (5)	12 (28)	9 (34)
	Ophthalmology	8 (16)	10 (10)	9 (27)	7 (10)	5 (12)	6 (22)
	Dental Surgery	4 (8)	15 (16)	8 (24)	1 (2)		1 (2)
	Otolaryngology	5 (10)	8 (8)	6 (18)	5 (8)	4 (10)	5 (18)
	Radiology****	6 (11)	3 (3)	5 (14)	14 (21)	12 (30)	13 (51)
	Gynecology	3 (5)	7 (7)	4 (12)	7 (11)	5 (13)	6 (25)
	Psychiatry	3 (5)	3 (3)	3 (8)	7 (10)	11 (27)	9 (37)
	Orthopedics	3 (6)	1 (1)	2 (7)	5 (7)	9 (21)	8 (30)
	Dermatology	3 (5)	2 (2)	2 (7)	7 (11)	2 (6)	4 (17)
	Urology	3 (6)		2 (6)	5 (7)	3 (8)	4 (15)
	Pediatrics	2 (3)	1 (1)	1 (4)	5 (7)	8 (20)	7 (27)
	Others*****	5 (9)	11 (11)	7 (20)	9 (14)	10 (25)	10 (39)
	Blank				1 (2)	2 (4)	2 (6)
The role in the latest clinical trial							
	As principle investigators	29 (57)			33 (50)		
	As co-investigators	46 (91)			44 (66)		
	As sub-investigators	22 (44)			13 (19)		
	As othres	2 (3)			8 (12)		
	Blank	1 (1)			2 (3)		
Experience in global clinical trial							
		12 (24)			41 (61)		
Interests							
	Drs who are interested in clinical trials	95 (186)	80 (83)	90 (270)	90 (135)	71 (172)	78 (310)
	Drs who are not interested in clinical trials	5 (10)	18 (19)	10 (29)	9 (14)	29 (70)	22 (87)
	Blank		2 (2)	1 (2)	1 (1)		0 (1)

*In total 7 (1 in KUHP and 6 in SUNU) doctors did not state their past experience in clinical trials.

**Internal Medicine in KUHP were hematology, cardiovascular medicine, gastroenterology, respiratory medicine, rheumatology, diabetic mellitus and nutritional medicine, nephrology, neurology, and geriatric medicine. Internal Medicine in SNUH were general internal medicine and immunology.

***Surgery in KUHP were gastrointestinal surgery, breast surgery, hepato-bilialy pancreatic surgery, transplantation surgery, neurosurgery, plastic surgery, cardiovascular surgery, and respiratory surgery. Surgery in SNUH were general surgery and cardiovascular surgery.

****Radiology in SNUH were radiology and radiation oncology.

***** Others in KUHP were Pathology, Emergency Medicine, Anesthesiology, Central clinical laboratory, and Transfusion Medicine & Cell Therapy. Others in SNUH were Pathology, Emergency Medicine, Family Medicine, Preventive Medicine, Clinical Pharmacology, and Rehabilitation Medicine.

Physicians with experience in clinical trials represented 196 out of 301 (65%) at KUHP and 150 of 398 (38%) at SNUH (Table 1). Overall, there was no major difference in the specialities and the respondents’ roles in previous clinical trials between the two university hospitals. Among doctors with previous clinical trial experience, 29% and 33% at KUHP and SNUH, respectively, had recent experience as principal investigators. 12% and 41% of doctors with clinical trial experience at KUHP (24/196) and SNUH (61/150), respectively, were involved in global clinical trials.

### Physicians’ interest in clinical trials

90% and 78% of all respondents at KUHP and SNUH, respectively, showed interest in conducting clinical trials (Table 1). Together a large proportion of physicians with previous clinical trial experience and many doctors without clinical trial experience expressed high interest in conducting clinical trials.

94% and 70% of all doctors at KUHP and SNUH, respectively, stated that they would be willing to participate or help (if time permits) in clinical trials organized by other doctors (Question #7).

### Time for informed consent

In Question #6, doctors with previous clinical trial experience stated how long it took them to obtain informed consent from study participants. In KUHP, 31% (60/196), 34% (67/196), 31% (61/196), and 3% (6/196) took <15 minutes, 15-30 minutes, 30-60 minutes, and >1 hour to obtain informed consent, respectively. In SNUH, 47% (71/150), 36% (54/150), 9% (15/150), 0% (0/196) took <15 minutes, 15-30 minutes, 30-60 minutes, and >1 hour to obtain informed consent, respectively.

### The merits of conducting clinical trials for doctors

**Figure 1:**
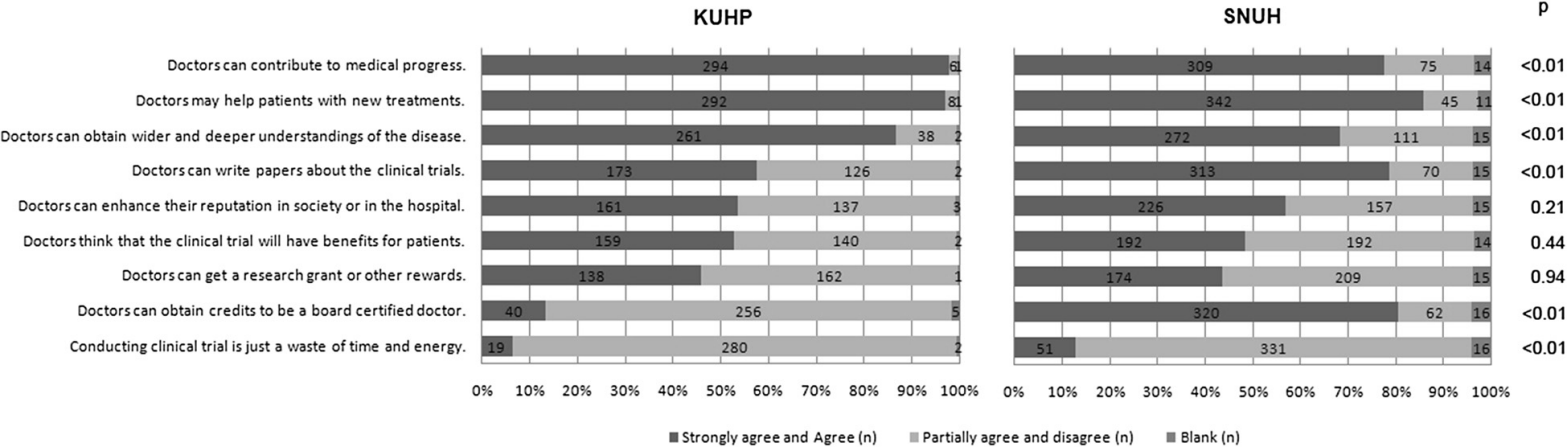
**The merits of conducting clinical trials for doctors.** (KUHP, n = 301; SNUH, n = 398). In Question #8 “What do you think are the merits of conducting clinical trials?”, the participants were asked to rate nine items by how strongly they agree using the following 4-tier scale: strongly agree, agree, partially agree, and disagree. The proportion of doctors responding as “strongly agree and agree” and “partially agree and disagree” in each institution was compared. 301 doctors at KUHP and 398 doctors at SNUH responded to the question.

In Question #8, the participants were asked to rate on a 4-tier scale 9 structured items that might be perceived as benefits for doctors in performing clinical trials. Additional comments were allowed to be entered by freehand. 301 and 398 doctors at KUHP and SNUH, respectively, responded to the question (Figure 1). At KUHP, the highest rated benefits (items rated as either “strongly agree” and “agree”) in decreasing order were: the contribution to medical progress (97%), helping patients with new treatments (97%), obtaining a wider and deeper understanding of diseases (87%), and the opportunity to publish their findings (57%). At SNUH, the highest rated benefits were: helping patients with new treatments (86%), obtaining credits for board certification (80%), the opportunity to publish their findings (79%), and the contribution to medical progress (78%). There was a significant difference between KUHP and SNUH in their opinions on performing clinical trials to obtain board certification, as this was least important determinant for doctors at KUHP (p < 0.01). 19 (6%) doctors at KUHP and 51 (13%) at SNUH thought that conducting clinical trial was just a waste of time and energy. No additional freehand comments were entered by any of the participants at KUHP or SNUH.

### Major problems faced by doctors in clinical trials

**Figure 2:**
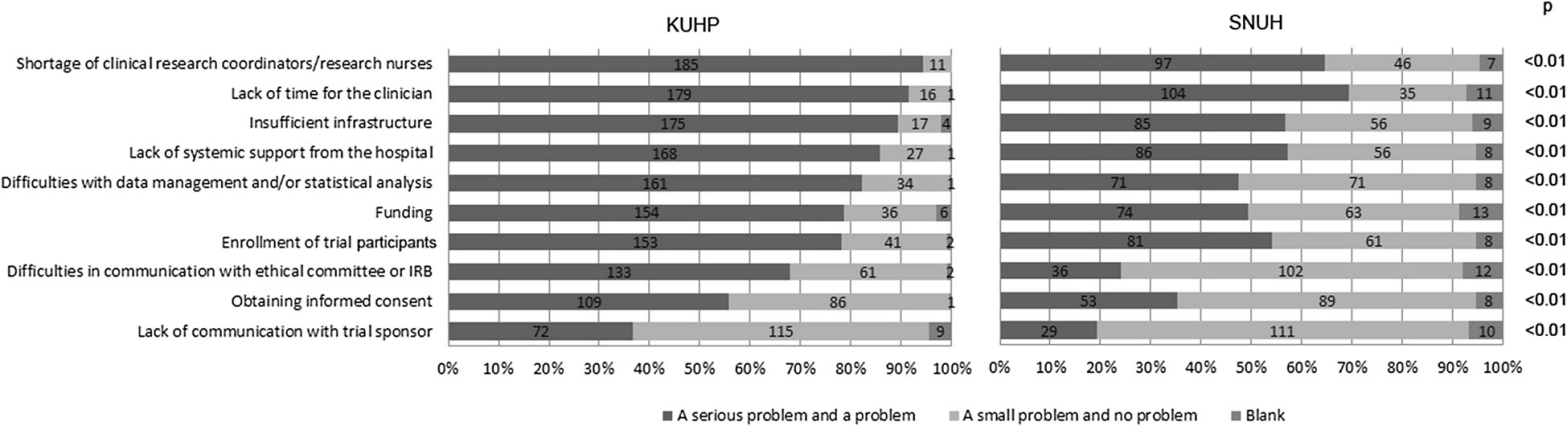
**Major problems faced by doctors with clinical trial experience (KUHP, n = 196; SNUH, n = 150).** In Question #10 “What are the major problems in conducting clinical trials?”, the participants were asked to answer the question according to the level of seriousness: a serious problem, a problem, a small problem, and no problem. The proportion of doctors responding as “a serious problem and a problem” and “a small problem and no problem” in each institution was compared. 196 doctors at KUHP and 150 doctors at SNUH with clinical trial experience responded to this question.

In Question #10, the physicians were asked about potential problems they faced in conducting clinical trials by rating 10 factors on a 4-tier scale. Among the subordinate items, 'systemic support from the hospital’ refers to personnel support such as research nurses, supporting entities and the general support environment provided to doctors not only for clinical trials but also for day-to-day clinical practice. This should be distinguished from the subordinate item 'infrastructure’ which refers to the specialized support system during clinical trials involving trained clinical trial professionals. We also distinguished 'enrolment of trial participants’ from 'problem with obtaining informed consent’ in items #7 and #8 of this question. 'Enrolment of trial participants’ refers to difficulties in patient recruitment because of safety concerns and the eligibility criteria. Difficulties in 'obtaining informed consent’ specifically related to difficulties in the interaction between the patient and the doctor. Additional comments were allowed to be entered by freehand. 196 and 150 doctors with clinical trial experience at KUHP and SNUH, respectively, responded to this question (Figure 2). The fraction of doctors rating the items as either “a serious problem” or “a problem” were: shortage of clinical research coordinators/research nurses (KUHP 94%, SNUH 65%; p < 0.01), lack of time (KUHP 91%, SNUH 69%; p < 0.01), insufficient infrastructure (KUHP 89%, SNUH 57%; p < 0.01), lack of systemic support from the hospital (KUHP 86%, SNUH 57%; p < 0.01), difficulties with data management/statistical analysis (KUHP 82%, SNUH 47%; p < 0.01), inadequate funding (KUHP 79%, SNUH 49%; p < 0.01), difficulties with enrolment of trial participants (KUHP 78%, SNUH 54%; p < 0.01), difficulties in communication with the ethical or IRB committees (KUHP 68%, SNUH 24%; p < 0.01), obtaining of informed consent (KUHP 56%, SNUH 35%; p < 0.01), and lack of communication with the trial sponsor (KUHP 37%, SNUH 19%; p < 0.01). No additional freehand comments were entered by any of the participants at KUHP or SNUH. Overall, doctors at KUHP appeared to encounter greater difficulties than doctors at SNUH, as over 70% of doctors at KUHP felt that they have limited resources including personnel (clinical research coordinators/research nurses, data manager and statistician), time, infrastructure, systemic support, and funding.

In Question #9, the physicians were asked about their experiences in clinical trials that underwent earlier termination than scheduled by the research protocol. The reasons of early termination were entered by freehand. 53 doctors at KUHP (27% of 196 doctors who have clinical trial experience) and 27 doctors at SNUH (16% of 150 doctors who have the experience) previously experienced an early termination of clinical trials. At KUHP, the reasons of the early termination of trials obtained by freehand were categorized and included the following in decreasing order of frequency: limited enrolment of trial participants (55%, 29/53), occurrence of adverse events (8%, 4/53), insufficient infrastructure (for example, limited access to imaging or testing devices) (6%, 3/53), negative analysis of interim results (4%, 2/53), transfer of doctors (4%, 2/53), difficulties in obtaining informed consent (4%, 2/53), shortage of staff (2%, 1/53), difficulties in long-term follow-up (2%, 1/53), and insufficient funding (2%, 1/53). The reasons at SNUH in decreasing order were occurrence of adverse events (26%, 7/27), limited enrolment of trial participants (19%, 5/27), interim analysis (15%, 4/27), at the sponsor’s request (15%, 4/27), protocol violation (11%, 2/27), publication of a similar clinical trial result by another institution (4%, 1/27), and unfeasible research design (4%, 1/27). There are many differences in the clinical trials conducted at SNUH and KUHP including their numbers, phases, designs, diseases, and the investigational agents (drug, device, biological) being tested. The higher fraction of adverse events observed at SNUH may be due to the higher risk profile of the trials there.

### Experiences and major obstacles faced in multinational clinical trials

**Figure 3:**
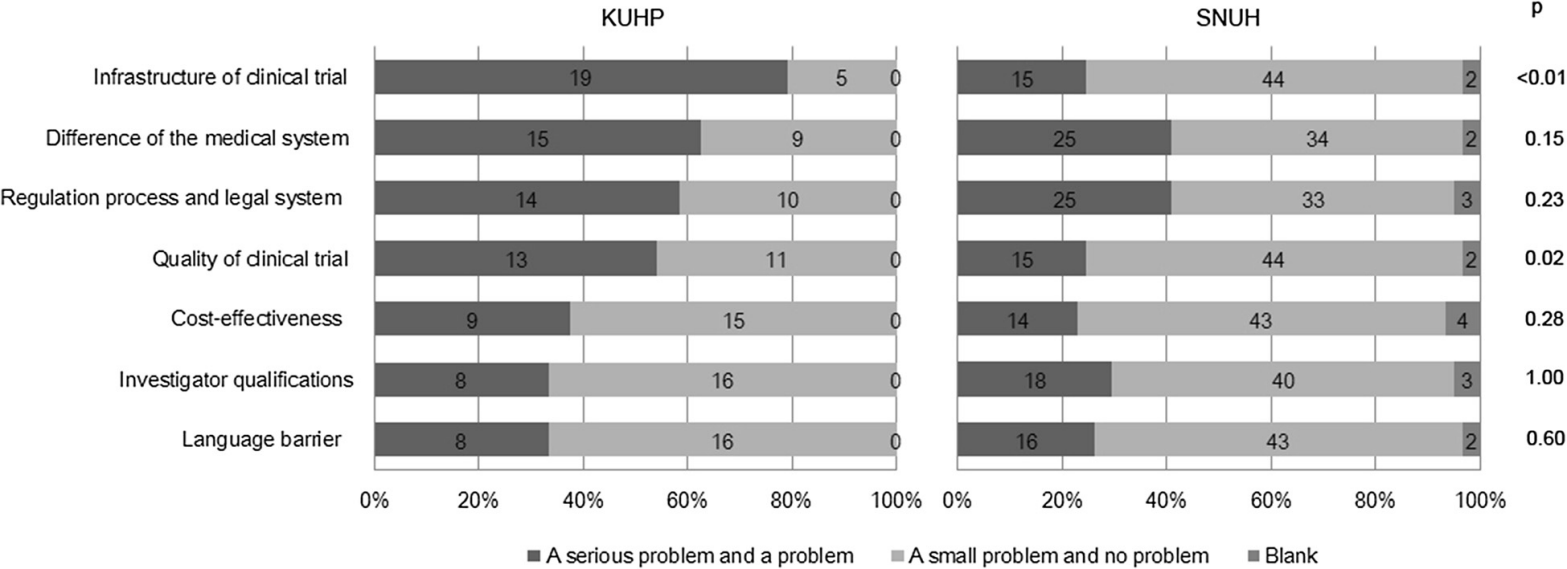
**Obstacles in multinational clinical trials.** In Question #16, the participants with global clinical trial experience were asked to rate 7 potential obstacles faced in a multinational clinical trial by their level of seriousness according to a 4-tier scale: a serious problem, a problem, a small problem, and no problem. The proportion of doctors responding as “a serious problem and a problem” and “a small problem and no problem” in each institution were compared. 24 and 61 doctors with global clinical trial experience at KUHP and SNUH, respectively, were answered in the question.

In Question #16, the participants were asked about their experiences in multinational clinical trials sponsored by a global pharmaceutical company. 12% (24/196) at KUHP and 41% (61/150) at SNUH of doctors with clinical trial experience were involved in such multinational clinical trials (Table 1). Of those who possessed global clinical trial experience, we further asked them to rate on a 4-tier scale 7 different potential obstacles that were faced in their participation in multinational clinical trials (Figure 3). Additional comments were allowed to be entered by freehand. 79% of global trial-experienced doctors at KUHP thought that the lack of infrastructure for clinical trials is most problematic, followed by differences in medical systems of different countries (63%), the regulation process and legal system (58%), and the quality of the clinical trial (54%). Cost-effectiveness, the qualifications of the investigator, and language barriers were considered obstacles by less than 50% of the multinational clinical trial-experienced doctors at KUHP. In contrast, only 25% of doctors with global trial experience at SNUH viewed the infrastructure and the quality of clinical trials as problematic. Moreover, only 41% of them thought that differences in the medical system and the regulation process and legal system were obstacles. Overall, <50% of global clinical trial-experienced doctors at SNUH considered any of the 7 items as obstacles. By freehand, one doctor in KUHP specified the difference in diagnostic criteria between countries as an obstacle that poses “a serious problem”.

### The knowledge of doctors at KUHP and SUNH regarding clinical trials

The participants were questioned whether they recognized the principles of the World Medical Association Declaration of Helsinki in Question #11 and were subsequently asked 4 true or false questions relating to the declaration in Question #12. Among all respondents, 97% and 74% of doctors at KUHP and SNUH, respectively, stated that they had knowledge of the declaration. 63% and 57% at KUHP and SNUH, respectively, answered all the questions related the declaration correctly.

**Figure 4:**
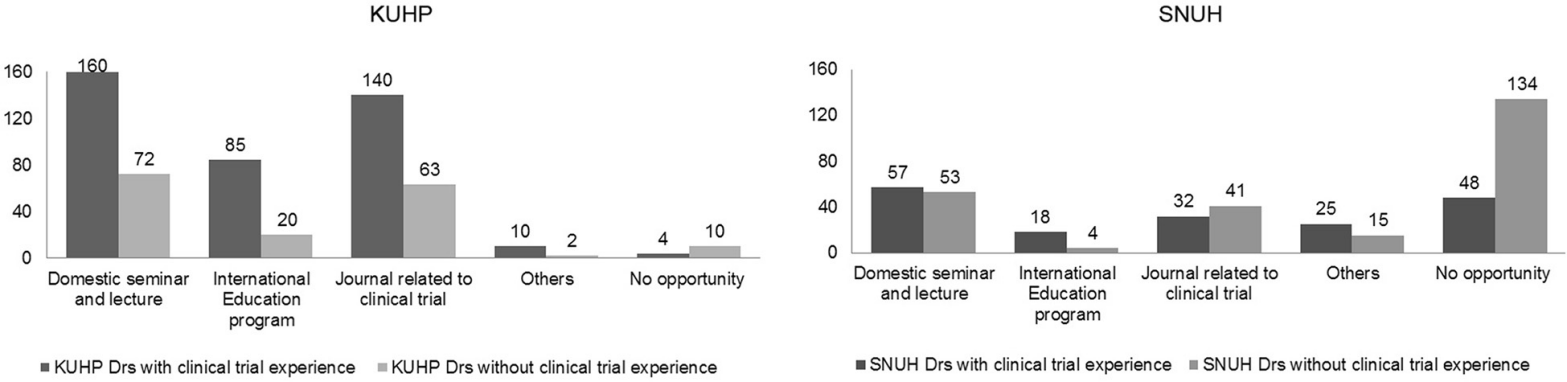
**Sources of information relating to clinical trials.** When asked about the place where they acquired knowledge related to clinical trials in question #13, 569 answers from 297 doctors at KUHP and 433 answers from 380 doctors at SNUH were obtained. 4 doctors (a doctor with clinical trial experience and 3 of doctors without clinical trial experience) at KUHP and 18 doctors (6 doctors with clinical trial experience, 11 doctors without experience, and a doctor who did not state their trial experience) at SNUH did not respond to the question.

When asked about their sources of information relating to clinical trials in Question #13, 569 answers from 297 doctors at KUHP and 433 answers from 380 doctors at SNUH were obtained. 283 and 198 doctors at KUHP and SNUH, respectively, answered that they have opportunities to acquire this information from various sources including domestic seminars and lectures, international educational programs, and clinical trial journals (Figure 4).

### The conditions and personnel required for overcoming the obstacles faced in clinical trials at KUHP and SNUH

**Figure 5:**
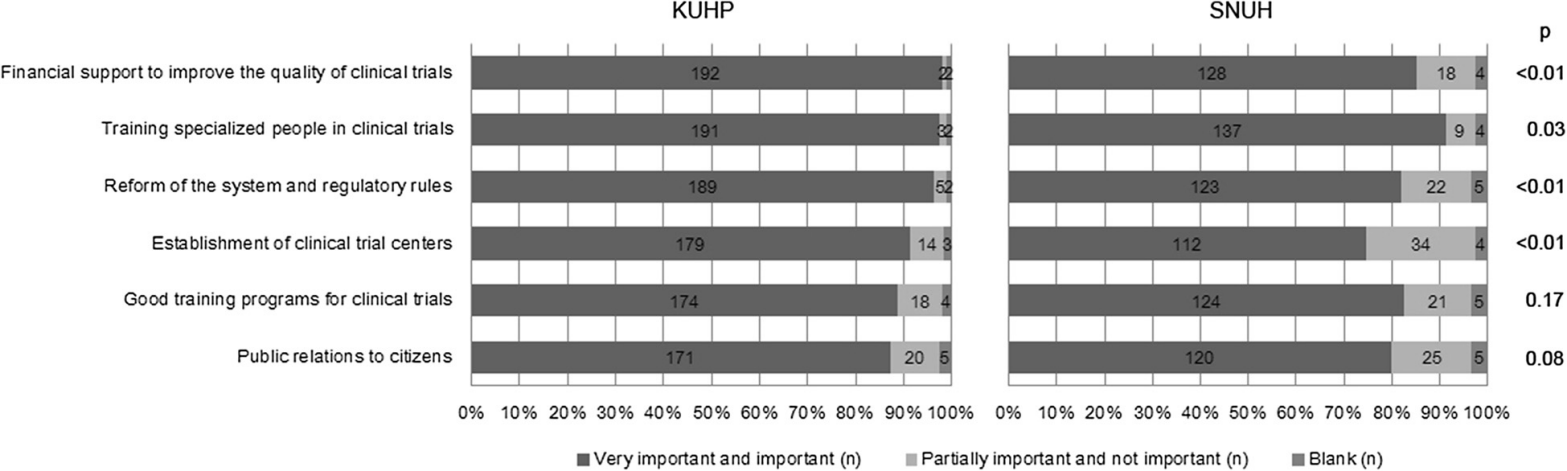
**Necessary conditions for overcoming the obstacles in conducting clinical trials at university hospitals.** In Question#14 “What do you think is important for overcoming the obstacles for the development of clinical trials?”, the participants were asked to rate 6 items by importance: very important, important, partially important, and not important. The proportion of doctors responding as “very important and important” and “partially important and not important” in each institution were compared. 196 doctors in KUHP and 150 doctors in SNUH with clinical trial experience responded to this question.

In Question #14, we asked the doctors to rate on a 4-tier scale 6 factors that were thought to be critical in overcoming the obstacles for the development of clinical trials. 196 and 150 doctors at KUHP and SNUH, respectively, who have clinical trial experience responded to this question (Figure 5). The fraction of doctors rating the items as either “very important” or “important” were: financial support to improve the quality of clinical trials (KUHP 98%, SNUH 85%; p < 0.01), training specialized people for running clinical trials (KUHP 97%, SNUH 91%; p = 0.03), reforming the system and regulatory rules (KUHP 96%, SNUH 82%; p < 0.01), establishment of clinical trial centers (KUHP 91%, SNUH 75%; p < 0.01), establishing good training programs for clinical trials (KUHP 89%, SNUH 83%; p = 0.17), and enhancing public awareness of clinical trials (KUHP 87%; SNUH 80%; p = 0.08).

**Figure 6:**
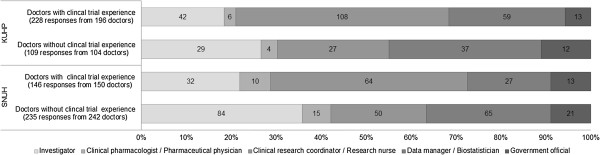
**Specialized training programs necessary for development of personnel.** In Question #15, among the 5 different types of personnel required for running clinical trials (investigators, clinical pharmacologists/pharmaceutical physicians, clinical research coordinators/research nurses, data managers/biostatisticians, qualified regulatory government officials), the doctors were asked who they thought the most effort into training should be placed. Answers from 196 doctors with and 104 doctors without clinical trial experience were obtained from KUHP. Answers from 150 doctors with and 242 doctors without clinical trial experience were obtained from SNUH.

In Question #15, among the 5 different types of personnel required for running clinical trials (investigators, clinical pharmacologists/pharmaceutical physicians, clinical research coordinators/research nurses, data managers/biostatisticians, qualified regulatory government officials), we asked the doctors who they thought the most effort into training should be placed (Figure 6). Clinical trial-experienced doctors at both university hospitals thought that the most important personnel were clinical research coordinators/research nurses: 47% of answers at KUHP (108/228) and 44% of answers at SNUH (64/104). However, doctors who did not have prior clinical trial experience at KUHP answered data manager and biostatistician as the most important (34%, 37/109 answers), and those doctors at SNUH answered principal investigator as the most important (36%, 84/235 answers).

### Additional open-ended comments from respondents

We welcomed additional comments by freehand in Question #17. 24 doctors out of 301 (8%) in KUHP and 13 doctors out of 398 (3%) in SNUH responded to the open-ended question. Among those who responded, 17 doctors at KUHP and 8 doctors at SNUH had clinical trial experience. A total of 41 comments from 24 doctors at KUHP and 15 comments from 13 doctors in SNUH were received. The comments were separated into 8 categories as shown in Table 2. In Question #17, KUHP doctors tended to provide negative comments about clinical trials focusing on problems with time constraints and the lack of support not only for clinical trials but also in daily clinical practice. In contrast, SNUH doctors tended to give more positive comments towards clinical trials, providing specific proposals for promoting clinical trials.

**Table 2:** Additional open-ended comments from respondents (N = 37)

		**KUHP**	**SNUH**
The number of responding doctors		24	13
Total number of comments		41	15
Categories			
C1	Lack of time, personnel support, and infrastructure	20	2
C2	Difficulties in recruitment of participants	13	6
C3	No benefit for individual doctors to engage in clinical trials	3	0
C4	Demand for education of investigators	2	1
C5	Necessity of clinical trials that fulfill the doctor’s academic interest	0	3
C6	Importance of funding	0	1
C7	Significance of global trials as a national strategy	0	2
C8	Criticism against this questionnaire	3	0

For example, in category C1, one KUHP doctor stated:

Neither coordinators nor nurses help us in the outpatient clinics in the university hospital with our usual clinical practice. We need to call patients, draw blood, and book the patient’s next visit ourselves. In addition, we don’t receive enough help in the inpatient ward either. Working under these terrible conditions, I don’t have any positive feelings to cooperate in clinical trials.

Similar comments relating to the lack of time, personnel support from the hospital, and infrastructure were noted in 9 out of 20 KUHP comments in category C1. There were also constructive comments in this category, which stated the necessity of clinical research coordinators (2), biostatisticians (4), better qualifications of reviewers in authorities (1), and improved infrastructure with sufficient personnel support (4).

In category C1, a SNUH doctor commented about the necessity of a data manager. Another SNUH doctor pointed out that the quality of the infrastructure varies widely among Korean sites:

The sites that can actually accomplish multicenter trials are rare and clinical trial infrastructures are fragile except for major large hospitals in Korea.

In category C2 of Question #17, 3 KUHP doctors mentioned difficulty in recruitment of patients. 2 KUHP doctors stated their reluctance to offer their help in randomized controlled trials because of their conflicting roles as clinicians versus investigators and its impact on their patient/doctor relationship. 4 KUHP doctors stated the patients’ reluctance to be recruited to clinical trials in Japan. A SNUH doctor mentioned difficulty in patient recruitment and recommended the establishment of a more efficient patient referral system to SNUH from other hospitals. Both KUHP and SNUH doctors agreed on the necessity of public relations for increasing recruitment of participants. However, a KUHP doctor raised concerns about a negative news report against clinical trials, which has discouraged doctors from participating in clinical trials.

In category C5 of Question #17, it was important to note that a SNUH doctor stated that clinical trials in South Korea tend to be motivated only by commercial interests. 2 other SUNH doctors stated their desire to conduct clinical trials that answer academic research questions. Similar comments were not obtained from respondents at KUHP.

In category C7, a SNUH doctor emphasized the importance of global trials as a national strategy in Korea and another SNUH doctor proposed that the generalized standard operating procedure acceptable by international standards should be developed for international audit.

## Discussion

### Main findings of the survey study

Doctors in both university hospitals, who participated in this study, expressed much enthusiasm in the execution of clinical trials. A large proportion of doctors at KUHP and SNUH already had past and/or present experience in conducting clinical trials. 12% and 41% of doctors with clinical trial experience also had experience in global clinical trials at KUHP and SNUH, respectively. The majority of doctors at both university hospitals believed that conducting clinical trials contributes to medical advances, which would ultimately lead to new and better treatments for patients. Time constraints and short-handedness of clinical research coordinators/research nurses represented the greatest obstacles in conducting clinical trials for doctors at both institutions. In general, compared to SNUH, a greater number of physicians at KUHP felt that there were more obstacles than incentives to conduct clinical trials.

### Social issues: lack of an adequate infrastructure, personnel, time, operational support, and public relations as an obstacle in running clinical trials

The lack of an adequate infrastructure was believed to be an obstacle for conducting clinical trials by 89% of all trial-experienced doctors and 79% of global trial-experienced doctors at KUHP. This was in contrast to SNUH, where only 57% of all trial-experienced doctors and 25% with global trial experience considered this a problem. Moreover, 91% of KUHP compared to 75% of SNUH trial-experienced doctors stated that the establishment of clinical trial centers would be important. The differences in views between the Japanese and South Korean doctors might be explained by the success of the South Korean government in creating the necessary infrastructure for conducting clinical trials such as the establishment of 15 Regional Clinical Trial Centers (RCTCs). KFDA allows clinical trials to be conducted only in the 150 KFDA-accredited hospitals and the RCTCs supported by the South Korean government are all large university hospitals capable of recruiting patients effectively for clinical trials. In contrast, the quality of hospitals in Japan is uniform, each with limited numbers of patients that are scattered throughout Japan, making patient recruitment into clinical trials difficult. This notion is consistent with the fact that more doctors at KUHP, compared to doctors at SNUH, viewed patient recruitment as a major obstacle in running clinical trials.

Doctors at both university hospitals thought enhancing public awareness of clinical trials was required for overcoming the obstacle. However, the lack of public relations is the issue in Japan. Saito et al reported that 50% of patients in general and 70% of all participants in clinical trials stated that the amount of information regarding clinical trials is scarce, and people perceive that the information services related to clinical trials to be insufficient in Japan [16]. Takita et al reported that many Japanese news reports emphasize the performance of pharmaceutical companies and incidents of misconduct, such as medication scandals, while providing insufficient information regarding clinical trials [17]. In fact, a doctor at KUHP in our study specifically commented on a recent negative news report on clinical trials, which has discouraged doctors’ from participating in clinical trials. Another possible reason for difficulties in enrolment of trial participants is the patients’ reluctance. Although both Japan and South Korea have a universal health insurance system with free medical access, differences in co-pays, reimbursement rates, and treatment regulations might make Japanese doctors more hesitant to offer patients to be recruited into clinical trials [18,19]. In the open-ended question of this survey, 4 KUHP doctors commented on the patients’ reluctance to be recruited to clinical trials in Japan.

Another major obstacle for doctors in conducting clinical trials was the lack of time. This is likely because of the shortage of doctors in Japan and South Korea. According to the World Health Organization data of World Health Statistics 2011, the number of physicians per 10,000 populations was 21 in Japan and 17 in South Korea, ranked at 59^th^ and 63^rd^, respectively out of 193 nations in the world [20]. These doctors need to cover the large number of hospital beds in Japan and South Korea, which are conversely ranked at 1^st^ and 3^rd^ in the world, respectively [20]. Similar problems were raised by Japanese doctors at KUHP in a survey conducted by Sumi et al [15]. Difficulties are compounded by the fact that most hospitals are not equipped with staff to streamline patient recruitment and manage the necessary paperwork, so the work continues to burden the already time-strapped doctors.

Nearly 80% of KUHP doctors with clinical trial experience thought that there was insufficient support from their hospital. However, at SNUH, only a little more than half of trial-experienced doctors believed that this was a problem. These findings were corroborated by additional comments from 9 KUHP doctors, who stated that there was a lack of personnel support from the hospital not only for clinical trials but also in their daily clinical practice.

A large majority of doctors from both institutes thought that the establishment of better training programs for running clinical trials is necessary to increase the number of skilled personnel. In addition, trial-experienced doctors at both KUHP and SNUH thought that they needed more clinical research coordinators/research nurses to conduct clinical trials more efficiently. The provision of sufficient study personnel or research nurses, who can regularly contact the participating physicians, might be the most effective intervention in overcoming this barrier.

The major obstacle other than infrastructure in participation in global trials for KUHP doctors was quality of the global clinical trial. 54% of KUHP doctors compared to only 25% of SNUH doctors stated that the quality of the clinical trial was an obstacle. This was unrelated to a potential language barrier that may exist in global trials, which was not perceived as an obstacle for many doctors at both KUHP and SNUH. Although the actual quality of clinical trials does not differ in both countries, the differing answers regarding quality may be explained by the fact that KUHP has less infrastructure, and fewer personnel and investigators who are experienced in multinational trials than SNUH. Because of their less experience in multinational clinical trials, KUHP investigators might be unaccustomed to the differences in the rules and regulations of document handling applied in global clinical trials compared to those in Japan. Therefore it is likely that KUHP doctors may be compelled to have more rigid perception in regarding the quality of trials or might have higher expectations when participate in multinational clinical trials.

### Pharmaceutical industry sponsored trial vs. academia initiated trial

There are two types of clinical trials which intervene with the clinical care of patients: development trials undertaken by pharmaceutical companies for the purpose of drug licensing approval, and post-approval studies mostly led by physicians/investigators. In trials conducted by drug companies, the scope of the study is limited as the trial is designed to obtain approval of their therapy for the indicated disease, which is dictated by regulatory requirements. Thus, this necessitates investigator-led post marketing trials for the advancement of medical science and treatment [21].

When conducting investigator-initiated trials in Japan, it is necessary to be compliant to The Ethics Guideline for Clinical Studies (EGCS) established by the Ministry of Health, Labour Welfare of Japan. The EGCS had been amended in July 2008 and was implemented in April 2009 to establish high-quality clinical study and evidence-building from clinical research findings [22]. When we conducted the questionnaire survey between September and December 2008, the level of interest for investigator-initiated trials and the EGCS was high amongst physicians [23]. It was likely that physicians at KUHP were feeling uncertain about changing their clinical trial environment due to the amendment in the guidelines at the time of the survey. Thus, the responses from KUHP doctors for this survey might reflect the issues relating academia led clinical trials rather than industry sponsored trials.

On the contrary, the greater proportion of doctors with global clinical trial experience at SNUH compared to KUHP possibly reflects the dramatic increase in global clinical trials in South Korea. Therefore, the responses from SNUH might reflect the issues in relation to those global trials. A SNUH participant in our study expressed her concern that clinical trials in South Korea tend to be motivated only by commercial interests, and 2 other SNUH doctors wished to conduct more clinical trials which aim to find answers to academic research questions.

### Awareness of issues surrounding clinical trials at the individual level

The process of obtaining informed consent was a huge burden for 55% of doctors at KUHP. In contrast, only 35% of doctors at SNUH felt that informed consent was a major obstruction in conducting clinical trials. We also found that doctors at KUHP took more time to obtain informed consent from study participants than those at SNUH. The difference might be explained in part by the higher proportion of Phase I trials at SNUH compared to KUHP, as the content of informed consent is different between Phase I trial and trials that have moved further along. There were 218 industry-sponsored trials in SNUH and ~20% of them were the Phase I trials, whereas of the 77 industry-sponsored trials at KUHP only 1 trial (1.3%) was a Phase I trial in 2008 at the time of the survey. There might be other factors as to why physicians face difficulties in obtaining informed consent of which one is “conflict of interest” [24,25]. Although our questionnaire did not specifically ask about opinions on potential conflicts of interest, 2 KUHP doctors commented on their reluctance to offer help to randomized controlled trials because of the difficulty in separating their conflicting role as a clinician versus an investigator, which might affect their patient/doctor relationship. In addition to conflicts of interests, a “defensive attitude” to avoid lawsuits may contribute to lengthening the time needed for informed consent. KUHP doctors may tend to explain everything about the clinical trial in an obsessive manner in their desire in not wanting to leave out anything unexplained. Cultural differences may also play a role in the increased time needed for obtaining informed consent. KUHP doctors may not acquire proficiency in obtaining informed consent because of the cultural gap between contractual relationships and affective trust [26,27]. Together, we believe that multiple factors could contribute to the perception that informed consent was a major obstruction in conducting clinical trials. However, in our study, we did not address any questions in matters relating to the deep personal feelings of physicians in obtaining informed consent, and thus we are not able to throw any light on the comparison between the opinions of physicians regarding this matter in both the university hospitals.

Although physicians faced many obstacles in conducting clinical trials, our results indicated that the majority of them from both university hospitals were quite willing to conduct clinical trials. This is in agreement with a previous survey in 2007 at KUHP, where 97% of all respondents believed that clinical research is necessary for physicians [15]. Compared to SNUH, doctors at KUHP showed a more cooperative attitude toward clinical trials operated by other doctors. The high interest level in clinical trials seen in physicians is reassuring, although it has been thought that university researchers in Japan prefer basic research to clinical studies [28].

As previously pointed out, physicians should be stimulated to feel a sense of commitment to medical innovation so as to enhance their participation in clinical research [29,30]. Doctors from both universities believed that the major benefits achieved from clinical trials were contribution to medical advances and the ability to provide new treatments to patients. While KUHP doctors were driven mainly by pure academic interest or for their desire to find new treatments for their patients, obtaining credits for board certification and co-authorship on manuscripts also served as motivation factors for doctors at SNUH. Board certification at SNUH allowed the doctors to be recognized as special experts and competent physicians in their medical specialties. However, these matters were not regarded as incentives for doctors at KUHP.

### The knowledge of doctors regarding clinical trials

Based on our questionnaire survey, we found that SNUH doctors possessed relatively less knowledge about the Helsinki declaration compared to KUHP and thus had fewer opportunities to obtain information about clinical trials. A possible reason for such a result may be attributed to the younger age of the respondents in SNUH compared to KUHP. Regarding Question #13 which relates to where one acquires information regarding individual clinical trial methods and results, we did not have a separate question asking about mandatory regulatory education and training related to execution of clinical trials. In addition, we had not incorporated any queries that could compare the type of educational seminars at KUHP and SNUH at the time of the survey. Consequently, we are unable to give an overview of the type of clinical trial educational training available at both university hospitals. Therefore, it is not possible for us to compare the results related to the existence or non-existence of knowledge related to clinical trials.

One of the positive outcomes of this survey is that SNUH went on to develop a mandatory course on GCP and research ethics for every medical student at Seoul National University Medical School in 2010. Furthermore, as of 2011 it is mandatory for every investigator including research staff and research nurses at SNUH to take at least one GCP course per year to conduct clinical research. Various types of clinical trial-related informational and educational material are now being provided to SNUH investigators through seminars, classes, pamphlets as well as educational websites since 2011 [31].

### Strengths and weaknesses of the present study

We believe our present study possesses several strengths. First, to our knowledge this is the first international survey designed to compare the attitudes of physicians toward clinical trials in Japan and South Korea. This was performed by concurrently administering an identical questionnaire survey for doctors at KUHP and SNUH, which are considered representative university hospitals in Japan and South Korea. Second, although there have been several studies reporting the views of physicians on specific clinical trials, there have only been a few studies that have conducted a survey about their general opinion on clinical trials in an university hospital setting. Third, this questionnaire was designed by the authors who support other doctors in conducting clinical trials in the academic research organizations of each university hospital, which allowed the survey to be focused on key issues regarding clinical trials. Therefore, the method of creating the questionnaire in this way itself was exploratory and has helped us to hone in on key issues surrounding clinical trials. Thus, through this survey, we believe that we have been able to identify the major issues that surround clinical trials conducted by physicians, which is an understanding vital for improving the clinical trial environment in both countries.

There were also a number of limitations to our present study. First, although both university hospitals are viewed as representative, the survey cannot wholly reflect the view of all doctors in both countries. Second, the differences in the response rate and the different composition of respondents in their age and position at KUHP and SNUH may have contributed to a selection bias. These differences could have posed as confounding factors when comparing the survey results from both countries in our study. Third, a selection bias exists whereby the results are skewed in the direction of those who were vocal about their opinions relating to clinical trials in both university hospitals. Fourth, the questionnaire was explained and handed out to staff by departmental directors, which could create social pressure to be active in clinical trials. Such professional and social pressure might lead physicians to respond to a survey in socially desirable ways. Fifth, the issues relating to clinical trial ethics were not covered by the questionnaire. While a knowledge test regarding the Declaration of Helsinki was provided, there were no questions on how doctors felt about possible conflicts of interest especially relating to randomised trials [32-34]. Finally, although using a structured anonymous questionnaire system helped in obtaining unbiased opinions from the doctors, this made it impossible to further explore in depth the individual merits and disadvantages regarding clinical trials for the doctors.

## Conclusions

Our results revealed that there might be approaches at two different levels to increase the activation of clinical trials. One is a social level approach involving establishment of better clinical trial infrastructure and providing sufficient clinical research professionals. The other is an individual-based approach targeted to increase the motivations of physicians toward participating in and conducting clinical trials. Both approaches would be necessary to maximally increase clinical trial activity.

## Abbreviations

GCP: Good clinical practice; ICH: International Conference on Harmonisation of Technical Requirements for Registration of Pharmaceuticals for Human Use; IND: Investigational new drug; J-GCP: Japanese GCP; KFDA: Korea Food and Drug Administration; K-GCP: Korean GCP; KoNECT: Korea National Enterprise for Clinical Trials; KUHP: Kyoto University Hospital; NDA: New drug application; PMDA: Pharmaceuticals and Medical Device Agencies; SNUH: Seoul National University Hospital.

## Competing interests

The authors declare that they have no competing interests.

## Authors’ contributions

TI participated in the study design and coordination, carried out KUHP survey, performed the data analysis, and drafted the manuscript. HJH participated in the study design and coordination, carried out the SNUH survey, performed data analysis, and drafted the manuscript. KOJ was on the steering committee at SNUH, participated in the study design, carried out the SNUH survey, and revised the draft of the manuscript. ES and TM conceived the study and participated in design and the questionnaire, and helped to draft the manuscript. SYK helped to carry out the SNUH survey and performed the data analysis. ST participated in the study design and performed statistical analyses. KN and TH participated in the creation of the questionnaire and the critical review of the manuscript from the view of qualitative research and psychology. EKC, KJC, MT and MM interpreted of data and revised the draft of the manuscript. MY was on the steering committee at KUHP and the organizer of this project. All authors have read and approved the final manuscript.

## Pre-publication history

The pre-publication history for this paper can be accessed here:

http://www.biomedcentral.com/1471-2288/13/130/prepub

## Supplementary Material

Additional file 1 Questionnaire. Click here for file
