# Supplementary material for: An international survey of physicians regarding clinical trials: a comparison between Kyoto University Hospital and Seoul National University Hospital
Source: BMC Med Res Methodol. 2013 Oct 25;13:130. doi: 10.1186/1471-2288-13-130 (PMC3830111; doi:10.1186/1471-2288-13-130)
Supplement: Additional file 1 — Questionnaire. [file 1471-2288-13-130-S1.pdf]

"An International attitude survey of physicians regarding clinical trials"

Please fill out the following questionnaire

Q1. How old are you?

- □ 1) < 29 years
- □ 2) 30-39 years
- □ 3) 40-49 years
- □ 4) > 50 years

Q2. What is your gender?

- □ 1) Female
- □ 2) Male

Q3. What is your position in your hospital?

- □ 1) Faculty（assistant professor, associate professor, professor）
- □ 2) Senior resident / staff doctor
- □ 3) Junior resident
- □ 4) MD, PhD course student
- □ 5) Others (Please Specify)

Q4. What is your specialty? Please, Specify ( )

Q5. Are you interested in conducting clinical trials?

□Yes, □No

Q6. Have you participated in any clinical trials before?

□Yes, □No

To those who answered [Yes]: What was your role in the latest clinical trial that you have participated in?

- □ 1) Principal investigator
- □ 2) Co-investigator
- □ 3) Sub-investigator
- □ 4) Others (Please Specify)

To those who answered [Yes]: How long does it usually take to obtain a patient's informed consent?

- □ 1) <15 minutes.
- □ 2) 15-30 minutes.
- □ 3) 30 minutes – 1 hour.
- □ 4) > 1 hour.

Q7. Would you like to participate in clinical trials organized by other doctors?

- □ 1) Yes I would.
- □ 2) I would like to help them if I have time.
- □ 3) No. I wouldn't, but I would like to know about the trial methods.
- □ 4) No, I wouldn't.

Q8. What do you think are the merits of conducting clinical trials?
Please check one box.

	I strongly agree.	I agree.	I partially agree.	I disagree.
1) Doctors may help patients with new treatments.				
2) Doctors can contribute to medical progress.				
3) Doctors can obtain a wider and deeper understanding of the disease.				
4) Doctors can obtain research grants or other rewards.				
5) Doctors can enhance their reputation in society or in the hospital.				
6) Doctors can obtain credits to be a board certified doctor.				
7) Doctors can write papers about the clinical trials.				
8) The clinical trial will have benefits for patients.				
9) Conducting clinical trial is just a waste of time and energy.				
10) Others (Please Specify)				

Q9 Have you ever participated in a clinical trial that was terminated before completing the expected schedule provided by research protocol?

- □ 1) Yes, I have had this experience.
  If yes, could you please describe the reasons why it was not completed?
  ( )
- □ 2) No, I haven't had this experience.
- □ 3) I don't know because I am conducting my first clinical trial now.
- □ 4) I have never conducted a clinical trial.

Q10. What are the major problems in conducting clinical trials?
Please check according to the level of seriousness.

	A serious problem	A problem	A small problem	No problem
1) Lack of time for clinicians				
2) Shortage of clinical research coordinators/ research nurses				
3) Difficulties with data management and/or statistical analysis				
4) Insufficiency of infrastructure				
5) Lack of systemic support from the hospital				
6) Difficulties in communication with the ethical committee or IRB				
7) Enrollment of trial participants				
8) Obtaining of informed consent				
9) Lack of communication with trial sponsor				
10) Funding				
11) Others ( )				

Q11. Do you know the “WORLD MEDICAL ASSOCIATION DECLARATION OF HELSINKI Ethical Principles for Medical Research Involving Human Subjects”?

- □ 1) Yes. I do. I appreciate it completely.
- □ 2) Yes. I mostly know it.
- □ 3) I know it to some degree.
- □ 4) No I don’t.

Q12. Which of the following statements are correct regarding medical ethics (indicate with a checkmark).

- □ 1) The subject matter of The World Medical Association Declaration of Helsinki is relevant to medical research on humans only and does not include human material or other identifiable data.
- □ 2) In medical research on human subjects, the interests of science and society should take precedence over considerations related to the well-being of the human subject.
- □ 3) The design and performance of each experimental procedure involving human subjects should be formulated in an experimental protocol. This protocol should be submitted for approval to an ethical review committee.
- □ 4) Negative as well as positive results should be published or otherwise be publicly available.

Q13. Where do you usually acquire information and knowledge related to clinical trials? Please check all that apply and describe the source title if applicable.

- □ 1) Domestic seminars and lectures (Title:)
- □ 2) International Education programs (Title:)
- □ 3) Journals related to clinical trials (Title:)
- □ 4) Others (Title:)
- □ 5) I don't have any opportunities to gain information.

Q14. What do you think is important for overcoming the obstacles for the development of clinical trials? Please check one box.

	Very important	Important	Partially important	Not important
1) Reform of the system and regulatory rules				
2) Financial support to improve the quality of clinical trials				
3) Training specialized people in clinical trials				
4) Establishment of clinical trial centers				
5) Good training programs for clinical trials				
6) Public relations to citizens				

Q15. The most effort should be placed in developing specialized training programs for which type of staff involved in clinical trials?

- □ 1) Investigator
- □ 2) Clinical pharmacologist / Pharmaceutical physician
- □ 3) Clinical research coordinator / research nurse
- □ 4) Data manager / Biostatistician
- □ 5) Qualified government official in the regulatory agency

Q16. Have you ever participated in a multinational clinical trial sponsored by a global pharmaceutical company?

□Yes, □No

To those who answered [Yes] : What are the major obstacles in participation in a multinational clinical trial? Please check according to the level of seriousness.

	A serious problem	A problem	A small problem	No problem
1) Investigator qualifications				
2) Quality of clinical trials				
3) Infrastructure of clinical trials				
4) Difference of the medical system				
5) Regulation process and legal system				
6) Language barriers				
7) Cost-effectiveness				
8) Others( )				

Q17. We welcome any additional comments.

Thank you for your time and cooperation.
